# Cardiac macrophages in maintaining heart homeostasis and regulating ventricular remodeling of heart diseases

**DOI:** 10.3389/fimmu.2024.1467089

**Published:** 2024-09-20

**Authors:** Mengjie Kang, Hui Jia, Mei Feng, Haolin Ren, Junjia Gao, Yueyang Liu, Lu Zhang, Ming-Sheng Zhou

**Affiliations:** ^1^ Science and Experiment Research Center, Shenyang Medical College & Shenyang Key Laboratory of Vascular Biology, Science and Experimental Research Center, Shenyang Medical College, Shenyang, China; ^2^ School of Traditional Chinese Medicine, Shenyang Medical College, Shenyang, China; ^3^ Department of Radiology, The First Affiliated Hospital of Nanjing Medical University, Nanjing, China; ^4^ Department of Cardiology, Second Affiliated Hospital, Shenyang Medical College, Shenyang, China; ^5^ School of Pharmacy, Shenyang Medical College, Shenyang, China

**Keywords:** macrophages, heart diseases, metabolic reprogramming, polarization, cardiac remodeling

## Abstract

Macrophages are most important immune cell population in the heart. Cardiac macrophages have broad-spectrum and heterogeneity, with two extreme polarization phenotypes: M1 pro-inflammatory macrophages (CCR2^-^ly6C^hi^) and M2 anti-inflammatory macrophages (CCR2^-^ly6C^lo^). Cardiac macrophages can reshape their polarization states or phenotypes to adapt to their surrounding microenvironment by altering metabolic reprogramming. The phenotypes and polarization states of cardiac macrophages can be defined by specific signature markers on the cell surface, including tumor necrosis factor α, interleukin (IL)-1β, inducible nitric oxide synthase (iNOS), C-C chemokine receptor type (CCR)2, IL-4 and arginase (Arg)1, among them, CCR2^+/-^ is one of most important markers which is used to distinguish between resident and non-resident cardiac macrophage as well as macrophage polarization states. Dedicated balance between M1 and M2 cardiac macrophages are crucial for maintaining heart development and cardiac functional and electric homeostasis, and imbalance between macrophage phenotypes may result in heart ventricular remodeling and various heart diseases. The therapy aiming at specific target on macrophage phenotype is a promising strategy for treatment of heart diseases. In this article, we comprehensively review cardiac macrophage phenotype, metabolic reprogramming, and their role in maintaining heart health and mediating ventricular remodeling and potential therapeutic strategy in heart diseases.

## 1 Introduction

Macrophages are an important type of immune effector cells, which inhabit almost all tissues and organs of the body, including the heart. Macrophages play important roles not only in tissue immunity and inflammation, but also in organ development, homeostasis and remodeling (1–3). The broad-spectrum of macrophages depends on their heterogeneity and plasticity, and macrophages can modify their phenotype or polarization, adapting to their microenvironment (4). The phenotype of macrophages manifests as two extreme polarizations: Classically activated M1 pro-inflammatory macrophages, also known as pro-inflammatory M1 macrophage, and alternatively activated M2 anti-inflammatory macrophages. M1 macrophages are involved in the initiation and maintenance of inflammation, recruiting other immune cells into inflamed tissue, and releasing inflammatory cytokines to kill foreign pathogens or microorganisms. M2 macrophages promote the resolution of inflammation, engulf apoptotic cells, drive collagen deposition, coordinate tissue integrity, and release anti-inflammatory mediators (5, 6). The delicate balance between M1 and M2 macrophages is crucial for maintenance of homeostasis. The imbalance of the two subtypes of macrophage may lead to many diseases including cardiovascular diseases, metabolic diseases and cancer (1).

Increasing evidence has shown that cardiac resident macrophages (CRMs) importantly contribute to the maintenance of heart health and the development of heart diseases (7). The origin of CRMs can be from the yolk sacs during embryonic development or hematopoietic cells-derived monocytes (8). Embryonic CRMs exist from embryonic development and persist into adulthood, and are mainly involved in heart development, homeostasis and repair (9). Upon the stimulation of inflammation or in the situation of heart diseases, the monocytes-derived macrophages are recruited, and CRMs are expanded in the heart to combat the injury stimuli and induce heart remodeling (10). Similar to macrophages residing in other tissues, CRMs can change their phenotype or polarization and undergo metabolic reprogramming in response to physiological or pathophysiological alterations in their microenvironment (11). Polarized macrophages exhibit metabolic changes, such as glycolysis, oxidative phosphorylation (OXPHOS), fatty acid oxidation (FAO), and amino acid metabolism. In addition to infectious diseases, macrophage polarization and metabolic programming also participate in the pathogenesis of cardiovascular diseases, especially heart ventricular remodeling diseases (12). This article focuses on reviewing the progress on the CRMs in maintaining heart health, mediating heart ventricular remodeling-related diseases and potential therapeutic strategy for heart diseases.

## 2 Macrophage polarization and metabolic reprogramming

**Figure 1:**
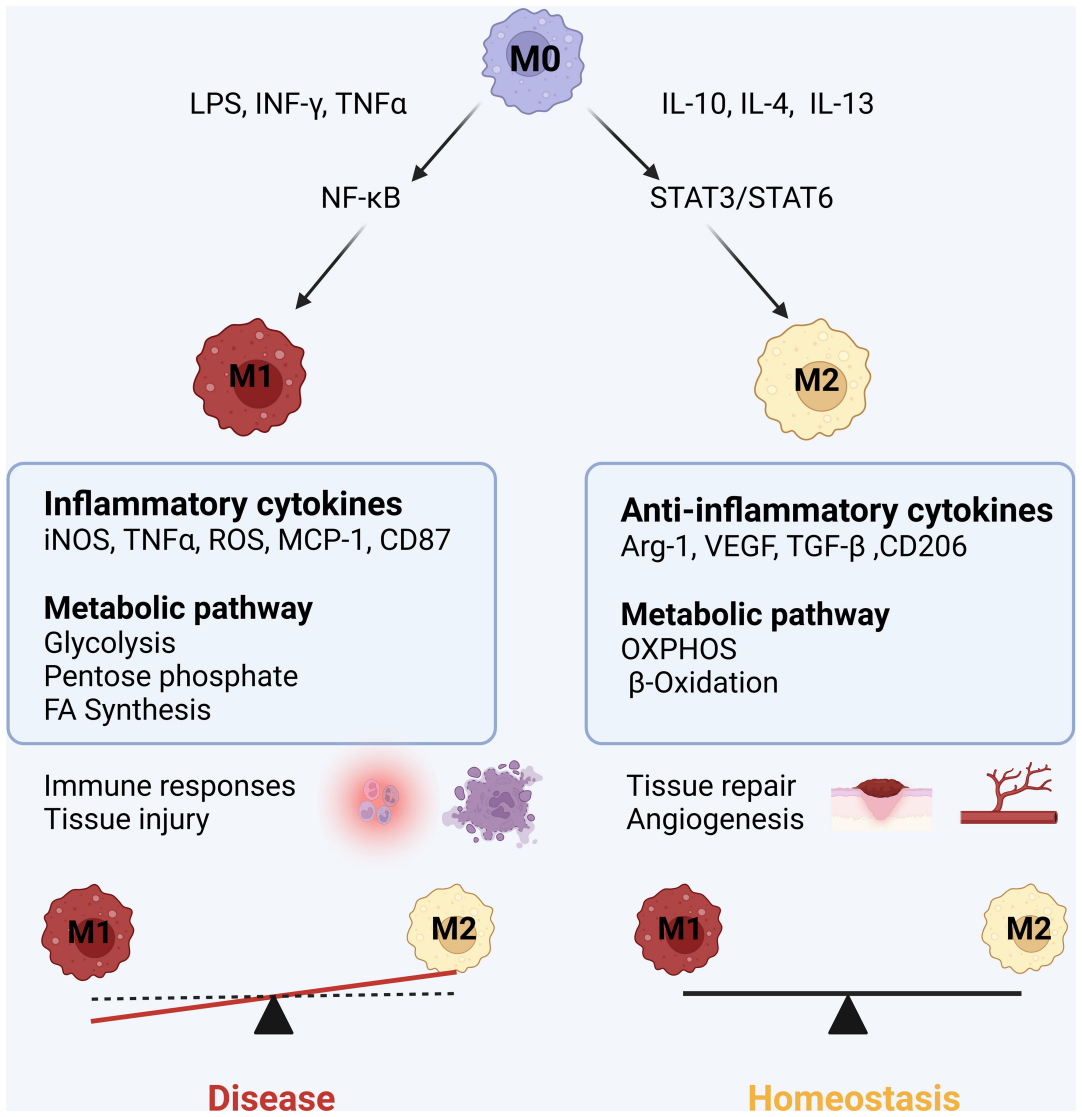
M1 and M2 macrophage polarization: Upon inflammatory (LPS, INFγ, TNFα) stimuli, macrophages are polarized into inflammatory M1 phenotype by activating NF-*k*B inflammatory pathway, simultaneously, the metabolic pathway of M1 macrophages swifts into glycolysis and pentose phosphate pathway. M1 macrophages release inflammatory mediators including inducible nitric oxide synthase (iNOS), reactive oxygen species (ROS), reactive nitric species (RNS), tumor necrotic factor (TNF) α, interleukin (IL)-1β, IL-6, monocyte chemokine protein (MCP)-1, leading to cytotoxicity and tissue injury. When inflammatory stimuli subsides or anti-inflammatory cytokines IL-10, IL-4 and IL-13 increase, macrophages are polarized into M2 anti-inflammatory macrophage associated with the switch of metabolic pathway into oxidative phosphorylation (OXPHOS) and fatty acid b-oxidation. M2 macrophages release arginase 1 (Arg1), VEGF, IL-10 and TGF-β1, promoting to tissue repair and wound healing.

Macrophages hold extreme plasticity and heterogeneity, and they can quickly adapt to the environment through modifying their phenotype or polarization (13). Unstimulated macrophages are under a quiescent or non-polarized state, which is also called M0 macrophage. Upon immune challenge, M0 macrophages have the ability to polarize into two distinct cell phenotypes of M1 inflammatory macrophages or M2 macrophages with the possibility to switch from M2 to M1 macrophage. M1 macrophages are typically activated by lipopolysaccharides (LPS), tumor necrosis factor (TNF)α or interferon (IFN)γ (14). M1 macrophages support a glycolytic activity to rapidly produce adenosine triphosphate (ATP) and efficiently fuel the cell at early stage of infection (15). The metabolic reprogramming toward aerobic glycolysis is called the “Warburg effect”, which is similar to metabolic reprogramming in cancer cells (16). Activated M1 macrophages produce large amounts of inflammatory cytokines and reactive oxygen species (ROS), which are beneficial for killing invaded microorganisms and controlling inflammation (17). However, the released inflammatory cytokines and ROS from M1 macrophages may also cause tissue chronic inflammation and injury, which is often present in cardiovascular diseases. M2 macrophages are usually induced by IL-4, IL-13 or transforming growth factor (TGF)-β (1). M2 macrophages have an intact tricarboxylic acid (TCA) cycle and use mitochondrial OXPHOS and FAO to generate ATP (10, 18, 19). M2 macrophages are involved in clearing apoptotic cells, driving collagen deposition and angiogenesis, and releasing anti-inflammatory mediators for anti-inflammatory and promoting tissue repair process (20). In addition, macrophages may have immune memory function on pathogen stimulation. M1/M2 macrophage polarization is always linked to the metabolic reprogramming of glucose, FAO, and amino acid metabolism (**Figure 1**).

### 2.1 M1 macrophage polarization and metabolism

Macrophages have surface and cytosolic sensors, such as toll-like receptors (TLRs) on cell surface and NOD-like receptors (NLRs) in the cytoplasm, which can respond to foreign pathogens or microbial products to trigger immune responses (21, 22). For example, LPS binds to TLRs on the macrophage surface, which subsequently activates downstream molecules to induce the activation of nuclear factor (NF)-*κ*B pathway and release inflammatory cytokines, such as IL-1β, IL-6, chemokine ligands (CXCL)9, CXCL10 and monocyte chemotactic protein (MCP)-1 (23, 24). Some pathogens may be engulfed by macrophages and bind to NLRs in the cytoplasm to form inflammasomes, which activate NF-*κ*B inflammatory pathway and promote macrophage activation (22). Interestingly, the stimulation of these sensors not only induce immune response but also drive macrophage into a Warburg-like response (25, 26). LPS-induced Warburg shift is a metabolic reprogramming that shares many similarities but differs significantly from the Warburg response in cancer cells. Cancer cells use glucose-6-phosphate as a fuel for lipid and nucleotide synthesis, and metabolize pyruvate to support amino acid and fatty acid synthesis for cell growth, while inflammatory responses mediated by TLRs produce the metabolites related to the antimicrobial activities, such as lactate, iNOS and ROS (26, 27).

Pro-inflammatory M1 macrophages shift their metabolism to aerobic glycolysis to meet the increasing energy requirement, and this metabolic adjustment is similar to the Warburg effect, referring to the metabolic reprogramming of macrophages, where macrophage rely solely on glycolysis to obtain energy, and the TCA cycle is inactivated even in the presence of oxygen (28). Glucose is metabolized through glycolysis to produce pyruvate and two molecules of ATP. Although glycolysis is relatively inefficient in ATP generation, the rate of glycolysis is very fast, the rapid increase in glycolytic metabolism is crucial for maintaining macrophage phagocytic activity (29). More importantly, increased aerobic glycolysis may compensate for the decreased ATP synthesis due to inflammation inhibition of mitochondrial respiration, which may help to maintain cellular ATP level and mitochondrial membrane potential (30).

**Figure 2:**
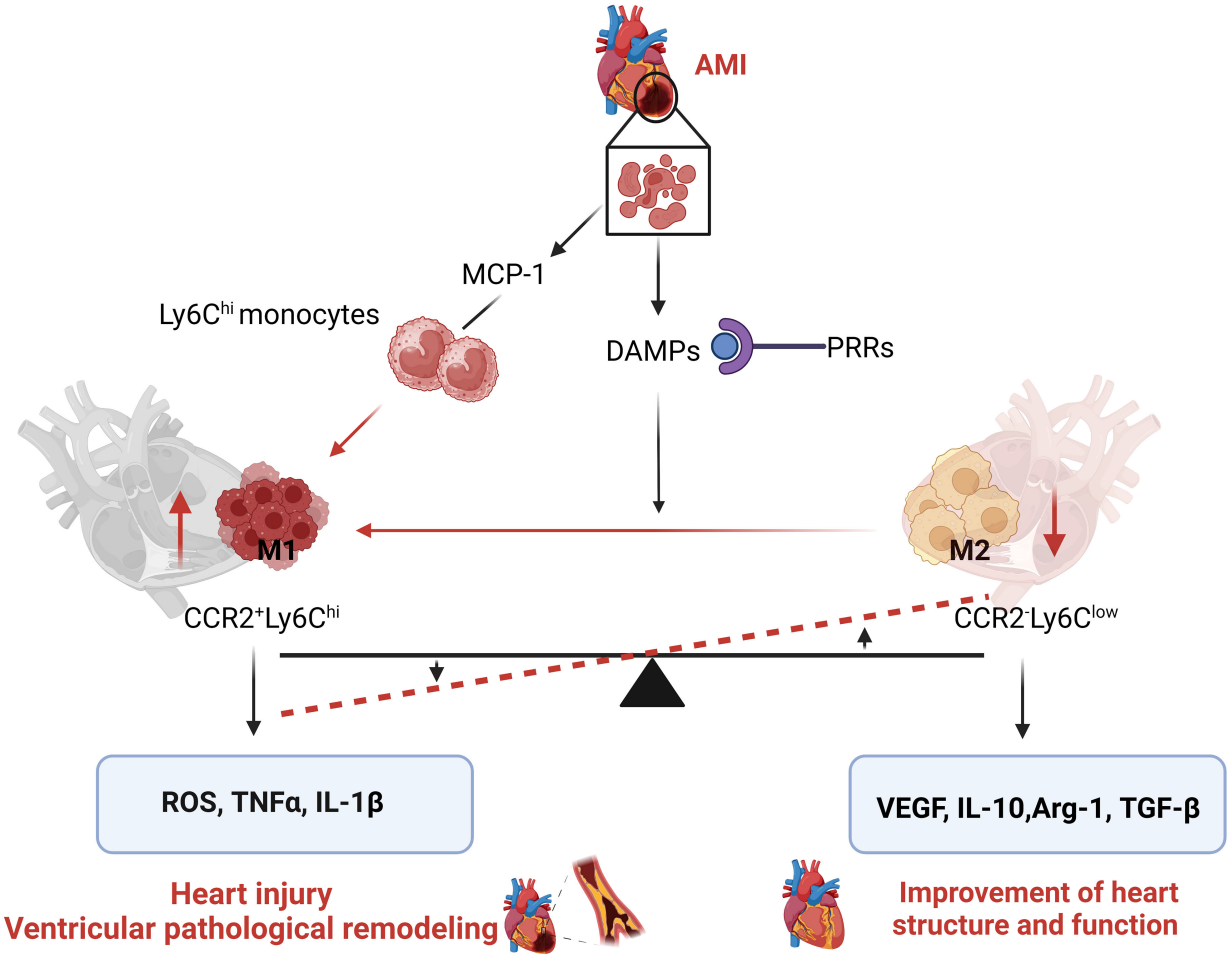
Metabolic reprogramming of M1/M2 macrophages: M1 inflammatory macrophages activate HIF1α pathway, which upregulates two key enzymes of glycolysis: 6-phosphofructose-2-kinase B (PFKFB) and pyruvate kinase M2 (PKM2), and increases activity of lactate dehydrogenase (LDH) that promotes the conversion of pyruvate to lactic acid, thus, increasing glycolysis. ATP generation from rapid glycolysis in turn inhibits mitochondria respiratory chain to reduce ATP generation from tricarboxylic acid cycle (TCA) through reduced key enzymes of TCA cycle, such as isocitrate dehydrogenase (IDH) and succinate dehydrogenase (SDH). The decreased activity of IDH and SDH increases the accumulation of substrates isocitrate and succinate in mitochondria which leak into the cytoplasm to continuously activate NF-*k*B and HIF1α, thus maintaining M1 macrophage activation and glycolysis. In other way, M2 macrophages have low level of glycolysis with intact TCA cycle, they generate ATP through mitochondria OXPHOS. In addition, M2 macrophages express high level of Arg1, which inhibit iNOS-derived NO production, which promotes the entry of pyruvate into the TCA cycle and inhibits glycolysis.

Glycolysis can provide metabolic intermediates for the synthesis of fatty acids, amino acids and ribose, which are necessary for cellular metabolic adaptation. In addition, during the glycolytic oxidative stage, NADP^+^ is converted into NADPH. NADPH can be utilized by NADPH oxidase to produce ROS, which activates redox sensitive transcriptional factor NF-*κ*B, promoting macrophage activation (31). In many circumstances, the activation of M1 macrophages is associated with hypoxic microenvironments, and hypoxia can profoundly affect macrophage function. Hypoxia inducible factor (HIF) 1α is an important transcriptional factor, which regulates a large amount of gene expressions, including the genes related to glycolysis and inflammatory mediators (32). For example, HIF1α upregulates glucose transporter (GLUT)1 to facilitate rapid uptake of glucose and lactate dehydrogenase to promote the conversion of pyruvate into lactate, which are crucial for metabolic glycolysis adaption of macrophage (33). HIF1α also upregulates pyruvate dehydrogenase kinase which inactivates pyruvate dehydrogenase to limit pyruvate into Krebs cycle. Therefore, HIF1α is an important regulator factor that promotes macrophage metabolic reprogramming and aerobic glycolysis (24, 34). The metabolic reprogramming of M1 macrophages mainly manifests by shutting down TCA cycle and shifting it into aerobic glycolysis, which helps M1 macrophages to maintain their inflammatory phenotype and produce pro-inflammatory cytokines and chemokines (**Figure 2**).

In glucose metabolism, two key enzymes are involved in regulating glycolysis and macrophage M1 polarization: 6-phosphofructose-2-kinase/fructose-2,6-bisphosphatase (PFKFB) and pyruvate kinase M (PKM)2, which are highly expressed in activated immune cells (35, 36). M1 macrophages mainly express PFKFB3 subtype and pyruvate kinase subtype 2. The PFKFB3 has a relatively low efficiency in catalyzing the conversion of 2,6-diphosphate fructose to 6-phosphate fructose; thereby increasing the glycolytic flux (37). PKM2 plays multiple roles in macrophage metabolism and polarization. In fact, when highly expressed, PKM2 exists in a balance between monomeric or dimeric enzymes without enzymatic activity and tetramers with enzymatic activity. Inactive enzymes of PKM2 monomer and dimer transfer to the nucleus and affect glycolysis by activating the expression of HIF1α gene, while tetramers with enzyme activity retains in the cytoplasm to promote glycolysis and M1 macrophage polarization (38).

It is well known that M1 macrophages are involved in the pathogens of cardiovascular diseases, especially atherosclerosis (1, 39). Atherosclerosis is considered as a chronic inflammatory vascular disease, and macrophages play a key role in the progress of atherosclerosis. Cluster of differentiation (CD)36 is an important scavenger receptor on the surface of macrophages, involved in the uptake of oxidized low-density lipoprotein (oxLDL), and oxLDL induces macrophage inflammation and activation (40). We have shown that nicotine can upregulate CD36 expression in macrophage which potentiates oxLDL-induced macrophage activation and foam cell formation (41).

### 2.2 M2 macrophage polarization and metabolism

M2 macrophages are induced by crucial cytokines, such as IL-4, IL-10, IL-13 and TGF-β, which are secreted by Th-2 immune cells (42). IL-4 and IL-13 induce M2 macrophage polarization by activating signal transducer and activator transcription (STAT)6 signaling, and IL-10 activates M2 macrophages by activating STAT3 (43, 44). M2 macrophages express high levels of CD206, CD163 and Arg1, and produce profibrotic factors TGF-β, vascular endothelium growth factor (VEGF) and insulin-like growth factor, therefore actively contributing to the resolution of inflammation, angiogenesis and tissue repair and healing. TGF-β, Arg1 and VEGF increase the production of collagen and angiogenesis, which favors tissue remodeling and wound repair (45). M2 macrophages have anti-inflammatory properties. In many cardiovascular diseases, activating M2 macrophages is considered to have protective effects. For example, it has been shown that activating M2 macrophages can increase the stability and regression of atherosclerotic plaques (46).

M2 macrophages fuel glucose and fatty acid, and rely on OXPHOS and FAO to generate ATP. Despite the controversy, it is generally believed that M2 macrophages are rarely dependent on glycolysis (12, 47). The mechanisms by which M2 macrophages exhibit low glycolytic activity are not fully understood. The following mechanisms may be related to low level glycolysis of M2 macrophages: Firstly, M2 macrophages have intact TCA cycle. They can provide ATP through mitochondrial OXPHOS (28). Next, PFKFB1 is a regulator of glycolysis, M2 macrophages have selective expression of PFKFB1, which can efficiently catabolize glycolytic activator fructose 2.6-bisphosphate to 6-phosphate, therefore limiting the glycolytic rate (29). Finally, M2 macrophages overexpress Arg1. As both Arg1 and iNOS use arginine as substrate, Arg1 may compete with iNOS for arginine substrate; thereby blocking iNOS-mediated NO production, which may reduce glycolysis (48) (**Figure 2**).

## 3 Cardiac macrophages in the maintenance of heart homeostasis

### 3.1 Cardiac macrophage lineage

The simplified definition of M1/M2 macrophages may fit in the situation of the *in vitro* experiments, and cannot accurately describe the characteristic of the cardiac macrophage phenotypes. Cardiac macrophages *in vivo* are more complex and heterogenous (49). Cardiac macrophage phenotype may be continuous in physiological and pathological states rather than just two types of polarized cells. CRMs have specific phenotypes and function in different cardiac regions and stages (50). According to the expressions of CCR2 and human leukocyte antigen DR (HLA-DR), human cardiac macrophages are classified into three different subgroups of CCR2^+^HLA-DR^lo^, CCR2^+^HLA-DR^hi^, CCR2^-^HLA-DR^hi^, and HLA-DR is a human homolog of the major histocompatibility complex (MHC)-II (51). According to the expression of the lymphocyte antigen 6 complex locus C (Ly6C) and MHC-II, mouse CRMs can distinguish four different phenotypes: CX3CR1^+^CCR2^-^Ly6C^-^MHC-II^-^ (MP1), CX3CR1^-^CCR2^-^Ly6C^-^MHC-II^-^ (MP2), CX3CR1^-^CCR2^+^Ly6C^+^MHC-II^-^(MP3) and CX3CR1^+^CCR2^-^Ly6C^-^MHC-II^+^ (MP4) macrophages (52). Based on the expressions of the biomarkers including lymphatic endothelial hyaluronic acid receptor (LYVE)1, phosphatidylserine receptor T cell immunoglobulin and mucin containing domain (TIMD)4, folate receptor β (FOLR2, TLF), CCR2, and MHC-II, Dick et al. (53) propose a more complex method of CRMs classification. They classify CRMs into three subgroups: 1) TLF^+^ CRMs expressing CX_3_CR1^lo^TIMD4^+^LYVE_1_^+^TLF^+^MHC-II^lo^CCR2^-^; 2) CCR2^+^ CRMs expressing CX3CR1^hi^CCR2^+^CD11c^+^MHC-II^hi^TIMD4^-^LYCR1^-^VE1^-^TLF^-^; 3) MHC-II^hi^ CRMs expressing CX3CR1^hi^MHC-II^hi^TIMD4^-^LYVE1^-^TLF^-^CCR2^-^. Among the group, CCR2^+^ CRMs have limited self-renewal ability and are constantly replaced by the circulating monocytes, TLF^+^ CRMs is almost entirely maintained by self-proliferation *in situ*, and MHC-II^hi^ CRMs only have a few subpopulations updated by monocytes. CCR2^+/-^ is considered as a key biomarker to distinguish between resident and non-resident CRMs which are conserved in humans, rats and mice, and TLF^+^CCR2^-^ and MHC-II^hi^ CRMs are considered CCR2^-^ CRMs (54).

With the development of novel biotechnologies, such as genetic fate mapping and lineage tracing, scientists can trace the origin of CRMs (9). It is generally believed that CRMs have two independent origins: One type of CRMs is established during the embryonic stage and embryonic development and can be self-sustaining and regenerating, this type of CRMs expresses CCR2^-^ and originates from yolk sacs, another type of CRMs expresses CCR2^+^ and originates from circulating monocytes, CCR2^+^ CRMs are continuously replaced by the circulating monocytes (55).

### 3.2 Macrophage regulation of heart electric activity and homeostasis

The generation and conduction of action potentials is an important feature of the heart. Under normal circumstances, the sinoatrial node generates action potential and controls heart electric activity, which propagate to all myocardial cells throughout the heart conduct system, inducing heart contraction (56). The heart contracts in the form of cardiomyocyte syncytium, and the synchronous contraction of the heart chambers is crucial for maintaining cardiac ejection function, and the completion of this function mainly depends on the high coordination of electric activity and contractile proteins in myocardial cells (57). It is well known that the conduction of cardiac electric impulses between myocardial cells is through gap junction (58). The simultaneous activation of action potentials in myocardial cells and an organized heart contraction require a normal gap junction communication (59, 60). Gap junctions are non-selective pores on myocardial cells, also known as gap junction channels, which allow ions and other small molecules to pass freely between myocardial cells, providing rapid conduction of electric impulse and electrical continuity between myocardial cells (61, 62). A single myocardial cell membrane only has gap junction hemichannels. These hemichannels converge at intercalated discs on myocardial cell membrane, which bind end to end with the hemichannels from adjacent myocardial cells, forming an intact gap junction channel (63).

The heart has four types of connexin proteins, including connexin (Cx)43, Cx45, Cx 40 and Cx30.2 (60, 64). Among them, Cx43 is the most abundant connexin protein, and is almost expressed in all parts of the heart except for the sinoatrial node. Cardiac gap junction intercellular communication is mainly controlled by post-translational regulation of Cx43. There are 21 phosphorylation sites at the C-terminal of Cx43, which can be phosphorylated or dephosphorylated by many protein kinases or phosphatases, thereby changing the localization of Cx43, the ion transport and electrical conductivity in gap junction (61, 65).

As mentioned above, CRMs are widely distributed in the heart. It has been shown that CRMs, which mainly express CCR2^-^, are also present in cardiac autorhythmic tissues, including sinoatrial node, atrioventricular node (AVN) and His-Bundle (66). Recently, Hulsmans et al. (67) demonstrated that CRMs, which were enriched in mouse and human AVN, had physical contact with neighboring conductive myocardial cells and facilitated electrical conduction in distal AVN and AV His-bundle through Cx43-containing gap junction. Using green fluorescent protein (GFP) labeled macrophages, they demonstrated that these macrophages are longitudinally distributed along the AV His-Bundle, with cytoplasmic portions extending and reaching long distance cardiac myocytes. Cx43-containing gap junction provides a structural framework for the interaction and communication between CRMs and conductive cardiac myocytes (68). Cell co-culture studies reveal a bidirectional regulation between macrophages and cardiac myocytes, and the depolarization of cardio myocytes induce the depolarization of macrophages, as only macrophages coupled with cardio myocytes generate depolarization potential (69). On the other hand, the electrical activity of macrophages, in turn, significantly affects the action potential of cardio myocytes, including reduction in the negative amplitude of resting potential, shortening action potential duration and refractory period, thus accelerating the electric conduction of cardio myocyte (68, 70). Cx43 plays an important role in electrical interaction and communication between CRMs and conductive myocardial cells, as the deletion of Cx43 in macrophages can eliminate the electrical interaction and communication between macrophage and myocardial cells (71). Furthermore, the conditional depletion of macrophage Cx43 or macrophage ablation can develop severe AV block in mice. These studies suggest that CRMs are required for the maintaining normal heart conduction in the AVN (67).

Although the mechanism by which macrophages participate in myocardial electrophysiology is not understood, Cx43 may be a significant medium that connects macrophages and conductive myocardial cells to regulate cardiac electrical activity. It has been shown that post-transcription regulation of Cx43 phosphorylation can alter gap junction function, affecting cardiac conduction (72). It has been shown that M1 polarization macrophage can upregulate potassium channel KCa3.1, which promotes Ca^2+^ influx in myocardial cells through Cx43, resulting in prolonged action potential durations (APD) (73, 74). In addition, the pro-inflammatory cytokines released from activated macrophage can affect cardiac electrical activity and induce cardiac electrical remodeling (75, 76). Thus, it tempts to hypothesize that CRMs may affect gap junction and cardiac electrical activity in myocardial cells through post-modification of Cx43 phosphorylation.

### 3.3 Macrophages maintain cardiac homeostasis

Macrophages are the most abundant and important immune cells in the heart, and play a critical role in heart development, maintenance of physiological homeostasis and induction of pathological ventricular remodeling in the heart diseases (9). Like macrophages in other tissues, CRMs have heterogeneity and functional plasticity to adapt to their microenvironment and meet the needs of their home tissue (12). CCR2^-^ CRMs are the main population, and expanded through self-renewal and proliferation *in situ* in the health heart, upon inflammatory or injury stimuli, the circulating monocytes can migrate into cardiac tissue and differentiate into cardiac macrophages, thus cardiac macrophages can be rapidly replenished by migrating peripheral monocytes to deal with the changed environment (7). These cardiac macrophages express CCR2^+^ and have strong phagocytic activity.

One mechanism by which cardiac macrophages maintain cardiac homeostasis may be through efferocytosis (77). Efferocytosis is a process by which macrophages recognize and engulf the dead cells, tissue debris or metabolic wastes by phagocytic receptor alone or in combination with other receptors, and is a characteristic function of macrophages. It is well known that myocardial cells are a type of long-lived and rarely self-renewal cells, myocardial cells have high metabolisms and are long-term exposed to intense mechanic stress, which may make cardiomyocytes produce lot of dysfunctional mitochondria and other cargos and eject them into the extracellular compartment (78). CRMs actively eat up these metabolic wastes to maintain cardiac homeostasis by efferocytosis. The depletion of CRMs may result in the accumulation of metabolic wastes and impair heart function.

CRMs maintenance of cardiac homeostasis also exhibit spatiotemporal coordination between CRMs heterogenous and local myocardial cell function (7, 9). It has been proposed that each heart chamber may have different CRM subsets with a specific phenotype and function. For example, the macrophages in the right atrium contain a distinct subset of CRMs that is much smaller than the CRMs in the ventricle tissue and almost non-existent in other chambers (79). In addition, CRMs between the atrium and the ventricle are also significantly different, which may be related to the differences in physiological functions, such as electrophysiological activity and contraction characteristics between atrial and ventricular muscle cells (80). These differences in function and structure between the atrium and ventricle may shape the heterogeneous CRMs subsets to adapt to their physiological function and local microenvironment.

## 4 The role of CRMs in ventricular remodeling in heart diseases

### 4.1 Myocardial infarction-induced ventricular remodeling

Myocardial infarction (MI) is a clinical syndrome of acute myocardial ischemic injury caused by partial or complete occlusion of the coronary artery (81). Persistent ischemia/hypoxia induces a series of alterations, including endoplasmic reticulum stress, ROS production, mitochondrial dysfunction, inflammation, cell death and ventricular fibrosis and remodeling. Postinfarction ventricular remodeling is the most prominent pathological alteration, which not only occurs in the region of infarcted myocardial tissue, but also reshapes whole ventricular structure and result in the progressive expansion of ventricle, and the development of HF and ventricular aneurysm (82, 83).

**Figure 3:**
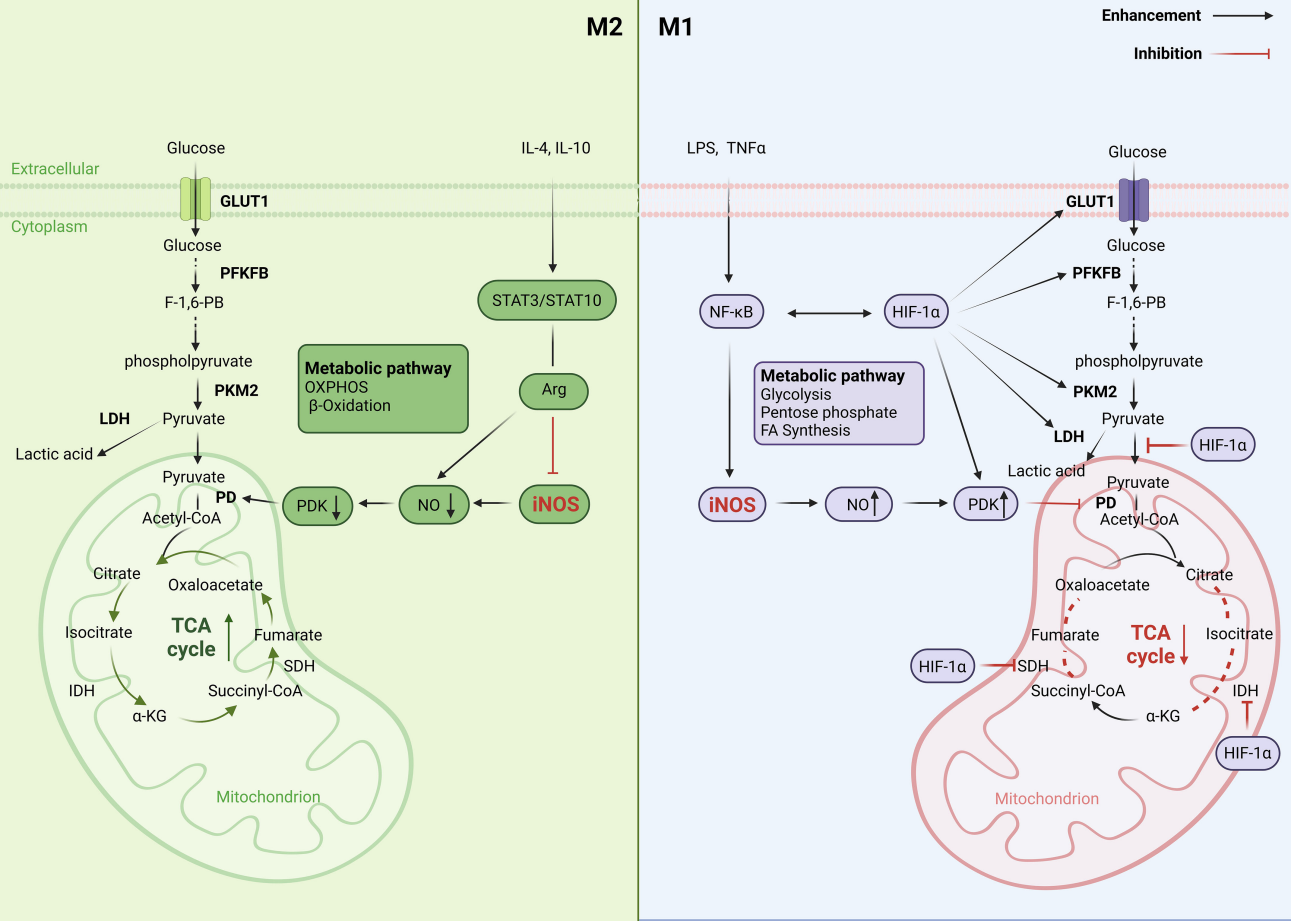
Acute myocardial infarction (AMI) alters cardiac macrophage subpopulation to induce pathological cardiac remodeling. After AMI, damaged or dead cardiomyocytes release MCP-1, which attracts circulatory Ly6C^hi^ monocytes to recruit to the infarcted area and differentiate into CCR2^+^Ly6C^hi^ macrophage. In other way, danger-associated molecular proteins (DAMPs) released from injury cells bind to pattern recognition receptors (PRRs) and promote the switch of CCR2^-^Ly6C^low^ cardiac resident macrophages (CRMs) into CCR2^+^ CRMs, resulting in the imbalance between CCR2^+^ CRMs and CCR2^-^ CRMs. CCR2^+^ CRMs secrete pro-inflammatory cytokines ROS generation to aggravate myocardial cell injury and death and induce pathological cardiac remodeling.

After MI, the heart initiates a serious response to repair damaged heart, and typically undergoes three phases: Inflammation, resolution of inflammation, and ventricular remodeling. Monocytes/macrophages are involved in all three phases and play a decisive role in these processes (84). In the early stage of MI, damaged cardiomyocytes release chemokines, such as MCP-1, C-C motif ligand (CCL) 2, CCL3. MCP-1 is an important chemokine that attracts bone marrow and spleen-derived Ly6C^hi^ monocytes to cross the vascular wall and migrate into the inflamed tissue, where Ly6C^hi^ monocytes differentiate into M1 inflammatory macrophage and gradually replace native CRMs (85). In addition, damaged cardiomyocytes can release danger-associated molecular proteins (DAMPs), which bind to pattern recognition receptors (PRRs) to form inflammasome to induce the activation of macrophages through activating the NF-*κ*B pathways (86). Activated M1 macrophages have potent phagocytic activity, which clear up the dead cells, damaged organelles and cellular debris, and help to maintain heart homeostasis and function (1). However, M1 macrophages can also secrete pro-inflammatory cytokines, such as IL-1β, IL-6, TNFα and increase ROS generation, which further aggravate myocardial cell injury and death, and worsen the microenvironment of myocardial cells, such as exacerbating hypoxia (5). Hypoxia activates the HIF1α pathway to induce the metabolic reprogramming of macrophages primarily through glycolysis (34, 38). If myocardial ischemia cannot be relieved, continuous inflammation and activated M1 macrophage are the main pathological mechanisms leading to the poor prognosis of MI (87). Approximately 4 days after MI, as inflammation subsides and inflammatory cytokine stimulation signals weaken, partial M1 inflammatory macrophages in the infarct area begin to transform into anti-inflammatory M2 macrophages (Ly6c^lo^) with a metabolic reprogramming of OXPHOS (88). M2 macrophages release cytokines, such as IL-10, vascular endothelial growth factor (VEGF), TGF-β, etc., which can inhibit inflammation, promote angiogenesis, collagen and extracellular deposition, and facilitate the reconstruction of heart structure and function (12). However, in most cases, myocardial ischemia at the infarcted area is not relieved, and the damaged and necrotic myocardial tissue serves as an inflammatory signal that continuously stimulates M1 macrophages to release inflammatory cytokines, resulting in an excessive inflammatory response, facilitating the adverse remodeling of myocardium (89). In addition, MI may impair macrophage efferocytosis to limit macrophage capability to clear up cellular debris and damaged organelle (77, 90) (**Figure 3**).

There are several molecules that can affect the polarization and metabolic reprogramming of macrophages, therefore affecting cardiac remodeling after MI. Ndufs4 encoded in mitochondrial complex I protein is involved in regulating cellular ATP synthesis. Cai et al. (91) have reported that specific KO of Ndufs4 in myeloid cells increase the pro-inflammatory cytokine expression of M1 macrophages, and delay the repair period after MI. In addition, the efferocytosis of macrophages in KO mice is impaired with the limitation of the proliferation and activation of fibroblasts in the infarction area, leading to adverse repair and ventricular remodeling. Leucine-rich repeating G-protein-coupled receptors (Lgr)4, also known as GPR48, is a member of the Lgr family that is gaining attention as a regulator of macrophage-related immune responses (92). Huang et al. (93) have shown that Lgr4 is highly expressed in activated macrophages, and specific knockout of Lgr4 in myeloid cells significantly improved cardiac function and cardiac remodeling in MI mice associated with reduction in pro-inflammatory Ly6C^hi^ mononuclear/macrophage in the infarct area.

Anti-inflammatory M2 macrophages play a crucial role in limiting adverse remodeling after MI. Zhang et al. (94) revealed that nucleophosmin (NPM)1 is a key regulatory protein that affects the transformation of anti-inflammatory macrophage M2 phenotype. Upregulation of NPM1 expression is closely related to poor cardiac prognosis in MI patients. Specific knockout of macrophage NPM1 can promote the transformation of M2 macrophage phenotype and improve cardiac function and tissue repair after MI. Marginal zone B and B1 cell-specific protein 1 (Mzb1) is an endoplasmic reticulum resident protein that plays an important role in immune response by enhancing the interaction of Ig weight chains with chaperone glucose-regulated protein (GRP)94 and increasing IgM secretion (95). We have recently shown that Mzb1 expression is significantly reduced in AMI mice, while Mzb1 overexpression can attenuate ROS production, inflammation, mitochondrial dysfunction and myocardial apoptotic cells, and improve cardiac function associated with reduced inflammatory macrophage infiltration (96). Considering the important role of Mzb1 in the regulation of inflammation and immune cells, we speculate that Mzb1 may promote cardiac repair after MI by facilitating reparative macrophage phenotype.

### 4.2 Ventricular remodeling with diabetes cardiomyopathy (DCM)

Diabetes mellitus (DM) is a metabolic disease characterized with increased blood level of glucose due to the impairment of insulin production and action. Long-term diabetes with uncontrolled high blood glucose often causes serious microvascular complications in vital organs, such as the heart, pulmonary and kidney (97–99). DCM is one of the most serious complications in the patients with diabetes. DCM patients have significant structural and functional abnormalities in the heart muscle as well as heart ventricular remodeling (100). These abnormalities include left ventricular hypertrophy and fibrosis, extracellular matrix (ECM) deposition, intracellular lipid accumulation with cardiac systolic and diastolic dysfunction (101).

The mechanisms by which DM promotes the development of DCM are complex and not fully understood. Multiple factors including hyperglycemia, hyperlipidemia, insulin resistance, ROS and inflammation contribute to diabetic heart injury and dysfunction (102). Among them, inflammation is absolutely necessary for the development and progression of DCM. Diabetes is a chronic low grade inflammatory disease, and metabolic disturbances, such as hyperglycemia and hyperlipidemia, stimulate immune cells infiltration into fat, vascular wall, the heart and kidney. These immune cells, especially macrophages, release inflammatory cytokines to initiate a low-grade inflammation in the infiltrated tissues or organs (103). Geng et al. (104) have reported that high glucose can directly induce macrophage activation and release M1 inflammatory cytokines through stimulating mitochondria ROS-dependent STING pathway *in vitro*. In the early stage of DCM, metabolic disturbance induces immune cell infiltration and chronic inflammatory response in the heart but does not significantly impair heart structure and function. However, continuous metabolic and inflammatory stress, especially inflammatory cytokines such as TNFα, IL-1β released from activated macrophages, could induce oxidative stress, endoplasmic reticulum stress, mitochondria dysfunction in the heart, leading to cardiomyocyte injury, apoptosis, cardiac fibrosis, eventually develop the advanced DCM (105).

Macrophages have been shown to play a critical role in the development and progression of DCM (105). In the early stage of diabetes, macrophages are recruited into adipose tissues. These recruited macrophages release inflammatory cytokines including TNFα, IL-1β and IL-6 to induce a local and systemic inflammation. The inflammatory cytokines inhibit insulin signaling pathway further to worsen systemic insulin resistance and metabolic disturbances (106). In addition, the inflammatory cytokines from systemic inflammation and released by infiltrated macrophages in the heart may initiate and exacerbate cardiac injury (107).

In addition to inflammatory cytokines, the impairment of macrophage phagocytosis may be another mechanism for DM development of DCM complication. Normally, macrophages eat up apoptotic cells and cellular debris by efferocytosis. It has been found that the phagocytic activity of macrophages in diabetic rat are impaired, which manifest low level of lysosomal enzymes and chemotactic activity in macrophages (108, 109). The impaired phagocytosis of macrophage is highly correlated with elevated blood glucose level, and the agents that lower blood glucose can improve macrophage phagocytosis (110). Thus, metabolic stress in DM may induce M1 macrophage activation to initiate cardiac inflammation to induce heart injury. On the other hand, hyperglycemia can also impair phagocytosis of M2 macrophages to impede clearance of apoptotic cells and cellular debris and the resolution of inflammation. Thus, the imbalance between M1 and M2 macrophages in microenvironment of heart tissue may importantly contribute to DCM (1, 106, 110, 111). The restoration of this balance may be an important therapeutic strategy for DCM. It has been shown that in streptozotocin (STZ) -induced diabetes models, the genetic deletion or pharmacological inhibition of chemokine receptor CCR2 suppresses M1 macrophage and slows down cardiac fibrosis. Additionally, metformin, the most popular medicine for clinical treatment of DM, has been shown to have selective inhibition of monocytes differentiation into M1 macrophages, and promotion of M2 macrophage to reduce inflammation and beneficial effects on the heart (112).

### 4.3 Heart failure

Heart failure (HF) refers to a clinical syndrome caused by various etiologies that impairs the structure and function of the heart, resulting in the heart being unable to pump enough blood to meet tissue metabolic needs (113). Based on ejection fraction, HF can be classified into two categories: HF with reduced ejection fraction (HFrEF) and HF with preserved ejection fraction (HFpEE). HFrEF exhibits cardiac systolic dysfunction, while HFpEF mainly presents cardiac diastolic dysfunction without significant reduction in ejection fraction (114). Although etiologies of HF are complex and heterogeneous, HF patients exhibit some common pathophysiological characteristics, such as the elevation of ventricular filling pressure and wall tension, and decreased myocardial contractile and ventricular compliance (115). Reduction in cardiac output may trigger systemic and local neurohumoral reflexes, immune response and other factors that cause the proliferation or apoptosis of myocardial cells, ECM deposition, inflammation, immune cell infiltration and myocardial fibrosis, resulting in ventricular pathological remodeling (11, 116, 117).

Regardless of the primary diseases of HF, inflammation and immune cell infiltration, especially monocytes/macrophage infiltration, play a pivotal role in the pathogenesis of HF (103). For HF caused by myocarditis or myocardial infarction, inflammation is the primary and essential factor to promote heart injury. Other types of HF, for example, hypertensive HF induced by aortic constriction, have significant increase in the infiltration of CCR2^+^ macrophages in myocardial tissue, as well as increased levels of local or circulating inflammatory cytokines, such as TNFα, IL-1β, IL-6 and MCP-1, suggesting that HF increases circulating monocyte-derived CCR2^+^ inflammatory macrophages in the heart (55, 118, 119). Cardiac macrophages have both beneficial and detrimental effects in the development of HF. On the one hand, CRMs, specifically CCR2^-^ CRMs can clear up the dead cells or cellular debris to maintain heart homeostasis through endocytosis (77, 78). On the other hand, CRMs release/produce inflammatory cytokines or ROS which induce myocardial cell injury, apoptosis and cardiac injury and fibrosis, resulting in pathological ventricular remodeling and further worsening heart dysfunction (120, 121). Heart fibrosis may also alter and reshape heart electrophysiologic activity, and become the pathological basis for the formation of arrhythmias. However, HF often shifts CRMs into inflammatory M1 macrophages and produces large amounts of inflammatory cytokines and chemokines associated with impairment of macrophage endocytosis (122). Clinical and experimental studies have shown that there is a significant correlation between plasma inflammatory cytokine level and the severity of HF. Inflammatory cytokines, such as TNFα and IL-6, have been used for biomarkers to predict the progression of HF (122, 123). Specific depletion of macrophage reduces cardiac inflammation, attenuates cardiac hypertrophy, fibrosis and ventricular function in animal models of HF.

It should be pointed out that the most current studies on inflammation, M1/M2 macrophages and HF are based on HFrEF (124). HFpEF exhibits more pronounced systemic inflammation and activated fibrotic pathway in the heart with increased profibrotic M2 macrophages. However, unlike anti-inflammatory beneficial effects of M2 macrophages in HFrEF, M2 macrophages in HFpEF exhibit more detrimental profibrotic effects. These M2 macrophages secrete large amounts of profibrotic factors such as TGF-β to enhance ventricular fibrosis, reduce ventricular compliance and impair ventricular relaxation (125, 126). Specific target macrophage phenotype is becoming a promising approach for treatment of HF, but it also faces many unsolved issues and enormous challenges.

### 4.4 Hypertensive left ventricular hypertrophy

Hypertension and its associated left ventricular hypertrophy (LVH) and HF remain a major challenge in the field of public health (127). Long-term high blood pressure causes disturb hemodynamic stress on the vascular and left ventricular wall, which promotes LVH hypertrophy, ECM deposition and fibrosis. Short-term ventricular hypertrophy and increased ECM is adaptive and may help to normalize ventricular wall stress and oxygen demand, which have compensatory effects on cardiac function (128). However, long-term ventricular hypertrophy, which lead to myocardium inflammation, fibrosis, cell death and development of HF, are unfavorable (129).

Increasing evidence has shown that the immune cells have been linked to the development and progression of hypertension and LVH (130). Long-term hemodynamic stress induces vascular and heart ventricular inflammatory responses that are increasingly recognized as key modulators of heart ventricular remodeling (131, 132). It has been reported that there is large number of monocytes and macrophages recruitment associated with increased expression of M1 macrophage cytokines in the heart and vascular wall of various hypertensive animal models (133, 134). Recruited macrophages release ROS and inflammatory cytokines, such as TNFα and IL-1β, inducing vascular and heart inflammation, cell proliferation and ECM deposition, and thus promoting heart ventricular remodeling (11). TNFα is an inflammatory cytokine mainly produced by macrophage. We have shown that deficiency in the TNFα gene can lower blood pressure, improve endothelial function, and attenuate left ventricular hypertrophy and fibrosis accompanied by reduced macrophage infiltration in the heart and vessel of deoxycorticosterone acetate (DOCA)/salt hypertensive mice (134). Kain et al. (135) have reported that depletion of monocytes/macrophages using clodronate liposomes reduces LVH by 17% and cardiac fibrosis by 75% in aortic constriction and pressure overload hypertensive mice. We used clodronate liposomes to deplete macrophages and found that clodronate liposomes reduce circulating monocytes by 70%, and display remarkable reduction in vascular and ventricular macrophages, lowering blood pressure, vascular and heart hypertrophy and fibrosis in both angiotensin (Ang) II hypertensive mice and Dahl salt-sensitive hypertensive rats (136, 137). These results suggest that macrophages in the heart and vessels play a critical role in hypertension and hypertensive heart remodeling (135).

Hypertension is often associated with increased circulating M1 inflammatory macrophages and expression of tissue M1 macrophage markers (138, 139). The mechanism of M1 macrophage activation in hypertension is not fully understood. The activation of the renin angiotensin system (RAS) has implicated in hypertension and hypertensive target end organ damage (140). Ang II is a pro-inflammatory mediator, Ang II promotes monocyte/macrophage infiltration into the vascular and heart wall. It has been shown that macrophages express all components of RAS (141). Recently we have shown that Ang II increases the expression of M1 pro-inflammatory cytokines TNFα, IL-1β, and iNOS in human THP1/macrophage, the underlying mechanisms are mediated by the activation of HIF1α/TLR4/NF-*κ*B signaling pathway, because siRNA HIF1α prevents Ang II-induced macrophage activation (133). Furthermore, we show that expression of angiotensin type 1 receptor (AT1R) is increased in the peritoneal macrophage of DOCA/salt hypertensive mice, and specific knockout of AT1R in myeloid cells attenuates macrophage recruitment in vascular wall and reduces heart and vascular hypertrophy and fibrosis without significant reduction in blood pressure (133). These results suggest that RAS may regulate macrophage phenotype and polarization, and dysregulation of RAS in immune cells may be an important mechanism of inducing macrophage activation and the vascular and heart remodeling in hypertension, which warrants further investigation.

### 4.5 COVID-19 infection-induced myocardial injury

COVID-19 originally surfaces as an acute viral respiratory syndrome caused by acute respiratory syndrome coronavirus 2 (SARS-CoV2) infection (142). However, severe COVID-19 is often associated with acute cardiac injury and heart ventricular remodeling, manifested arrhythmias, myocardial ischemia, ventricular dysfunction, recurrent decompensated HF, or myocarditis (143). The mechanism of heart injury caused by COVID-19 is complex and not yet fully understood. The cardiac complications of COVID-19 are heterogeneous and can present at any periods of the COVID-19 infection, even occur as a sequela of post-infection (144). The patients with a history of heart disease and risk factors are more susceptible to have cardiac complications of COVID-19, or develop into more severe illness, even die (145). Serum level of troponin, as a biomarker of acute heart injury, increases in the patients with severe COVID-19, and the serum concentration of troponin is significantly correlated with the severity of the disease and the risk of death in the patients with COVID-19 (146).

Cardiac autopsy from the patients with COVID-19 has shown that, unlike traditional viral myocarditis which mainly involves lymphocytic infiltration, there are more CD68^+^ cells in the heart of the patients with COVID-19, and CD68^+^ cells are widely distributed in the myocardium and epicardium of the heart, suggesting an important role of macrophages in COVID-19 infection-induced cardiac injury (147). In addition, high levels of TNFα, IL-6, and CCL2 inflammatory cytokines are detected in the circulating blood and the heart of patients with COVID-19, it is worth noting that these inflammatory cytokines are also important markers of M1 macrophage activation (148). These suggest that monocyte/macrophage infiltration might be more closely related to heart injury caused by SARS CoV-2 infection. Thus, some investigators propose that macrophages may play a more important role in myocarditis or cardiac injury caused by SARS-CoV-2 (149). Severe COVID-19 patients have long-term adverse heart remodeling, a group of autopsy data from the University of Alabama showed that 80% of the dead patients with severe COVID-19 had myocardial fibrosis, 72% had myocardial hypertrophy, and 66% had microembolization (150).

It is known that SARS-CoV2 enters host cells mainly through the interaction between angiotensin converting enzyme (ACE)2 receptor on the host cells and spike protein from SARS-CoV2 (151). After spike protein binds to ACE2 receptor on host cells, the virus is internalized and enters the cell, replicating DNAs within the host cells, which may disrupt the organelles, such as mitochondria and endoplasmic reticulum (152, 153). ACE2 is a component of RAS and plays an important role in the regulation of cardiovascular function. ACE2 receptor is widely distributed in the cardiovascular system (154). Thus, the ACE2 receptor may be an important target for SARS-CoV2 affecting the cardiovascular system. Unlike cardiac cells with rich ACE2 receptor, monocytes and macrophages have no or low level of ACE2 receptor, it is found that SARS-CoV2 enters and activates monocyte/macrophage mainly through GRP78 rather than ACE2 receptor (155). It is similar to the finding that is seen in macrophage activation syndrome: The activated macrophages in COVID-19 release large amount of inflammatory cytokines in their infiltrated organs, such as the lung and the heart, to trigger inflammatory storm, which may lead to fatal damage to the heart, lung and other vital organs (148, 156). Specific targets on macrophage phenotypes and reduction in the risk of the inflammatory storm may be a promising therapy for severe COVID-19 patients.

### 4.6 Heart valve diseases

Heart valve diseases are a common cardiovascular disease, which can be classified into congenital and acquired heart valve diseases (157). Due to significant alterations in cardiac hemodynamics within heart chambers, heart valve diseases often cause ventricular hypertrophy, fibrosis, and other ventricular remodeling as well as cardiac dysfunction (158). Myxomatous valve disease and calcific valve disease are the two most common types of heart valve disease. Myxomatous valve disease often invades the mitral valve, and the pathological changes mainly exhibit progressive extracellular matrix abnormalities and thickening of leaflets, which can ultimately lead to mitral prolapse and regurgitation (159). Aortic valve calcification is characterized by aortic stenosis and mineralization, which are more common in elderly and ischemic heart diseases (160).

Like myocardial tissue, there are multiple immune cells present in the development of heart valves, which account for approximately 8% of the total number of heart cells. Among them, 75% of cells express the biomarkers of macrophages (161). Macrophage lineages in the heart valve are heterogenous, including embryonic resident macrophages and macrophages derived from circulating monocytes. These macrophages play a critical role in heart valve development, physiological homeostasis, and pathological heart valve remodeling (162). During embryonic development, the heart valve is mainly composed of resident macrophages that express CCR2^-^. After birth, macrophages from the hematopoietic lineage start to recruit to the heart valve (161, 163). In neonatal heart valves, macrophages mainly express M2 macrophage marker CD206, while in myxomatous heart valves, recruitment macrophage expressing M1 inflammatory markers significantly increases. Interestingly, the time and number of macrophage recruitment to the heart valve are closely related to the pathological thickening of the myxomatous heart valve (164). It has been shown that the heart valves have already recruited with large numbers of inflammatory macrophages before the heart valve begins to become pathological thickness and heart functional damage. The depletion of macrophages prevents heart valve thickness (163). These results suggest that macrophages play an important role in pathological remodeling of heart valves, especially myxomatous mitral valves.

### 4.7 Atrial fibrillation and heart electric remodeling

Atrial fibrillation (AF) is the most common type of cardiac arrhythmia associated with a range of heart diseases, such as ischemic coronary heart disease and HF (165). AF is often accompanied with electric remodeling and structure remodeling in atrial myocardial cells (166). Electrical remodeling mainly manifests as changes in the number, distribution and functional activity of atrial ion channels and gap junction proteins, resulting in a shortened or prolonged effective refractory period (167). The characteristics of structural remodeling are progressive collagen deposition and fibrosis in the atrium (168). Electric remodeling is considered an important factor affecting individual susceptibility to AF, whereas structural remodeling is a pathological base for persistent AF and other arrhythmias (169).

The increasing evidence suggests a close correlation between AF and inflammation. It is found that the number of immune cells expressing CD45^+^ and CD68^+^ (a macrophage marker) significantly increases in the atrium of AF patients, except macrophages, other immune cells, including mast cell, dendritic cells and CD3^+^ T cells, are also increased in the patients with AF (170, 171). The patients with AF have higher plasma level of inflammatory cytokines TNFα, IL-1β and IL-6 (172). The immunotherapies that target on specific cytokines or their receptors have shown to exhibit therapeutic beneficial effects on AF. Colchicine is a broad-spectrum of anti-inflammatory drug that inhibits IL-1β production and TNFα-induced NF-*κ*B inflammatory pathway. COPPS trial shows that treatment with colchicine for one month lowered the incidence of postoperative AF compared to placebo (173). As we know that IL-1β is an important cytokine released by M1 macrophage, a clinical trial demonstrates that targeting on IL-1β by canakinumab can lower the incidence of AF recurrence after electrical cardioversion in the patients with heart diseases (174). In addition, two preclinical studies have shown that depletion of macrophages lowers the incidence of AF. These results strongly support the notion that inflammation and macrophages play an important role in the pathogenesis of AF (168). It should be noted that due to limited size of these clinical trials, the effectiveness of these anti-inflammatory drugs in treating AF still needs further validation through large-scale clinical trials.

Recent studies have shown that inflammation and immune system have great impact on atrial electric activity. As AF is always associated with cardiac chronic inflammation and immune cell infiltration, especially macrophages (168). It has been shown that inflammatory cytokines released by macrophages, such as TNFα, IL-1β, IL-6 and macrophage inhibitor factor (MIF), can affect the activity of various ion channels in the atrium and induce atrial electric remodeling (175). TNFα inhibits T-type calcium channel α1G subunit and sarcoplasmic reticulum Ca-ATPase that disrupt intracellular Ca_2_^+^ homeostasis in atrial myocytes, enhancing arrhythmogenic activity (176). Macrophage-derived IL-1β suppresses the expression of L-type calcium channel and decreases calcium channel expression, resulting in abnormal Ca^2+^ handling imbalance (76). Connexin proteins are important components of gap junction in myocardial cells and play a crucial role in the electric conduction of the heart, it has been shown that pro-inflammatory cytokines TNFα, IL-1β and MIF can inhibit the expression of Cx40 and Cx43, affecting electric activity and conduction in the atrium (177). Therefore, immune cells or macrophages induce atrial electric remodeling through direct or indirect manners, promoting the occurrence of AF.

## 5 Macrophage-based therapeutic strategies for heart diseases

Despite significant progress in treatment options, cardiovascular diseases, such as MI and chronic HF, remain the leading cause of death over the world. Chronic inflammation and its related irreversible fibrosis are the main underlying pathophysiological causes that promote heart diseases and ventricular remodeling (178, 179). Cardiac macrophages have been recognized as the main driving force behind the development of these pathophysiological conditions. Macrophage-based immunotherapies have been recommended and may bring new prospects for the treatment of ventricular remodeling in heart diseases (119).

### 5.1 Inhibition of macrophage recruitment and the depletion of cardiac macrophages

As discussed above, recruited monocyte-derived macrophages importantly contribute to heart inflammation and adverse heart remodeling in many cases of heart diseases (54). Thus, the strategies aiming at inhibition cardiac macrophage recruitment or the depletion of macrophages may be promising therapy for heart diseases. MCP-1, also known as CCL2, and its receptor CCR2 (CCL2-CCR2) axis is an important factor to induce the recruitment of monocytes and macrophages into the vascular wall and the heart (180). Recent several preclinical studies have shown that the inhibition of CCL2 through anti-CCL2 antibody or CCR2 depletion can attenuate adverse left ventricular remodeling in MI mice, and silencing CCR2 gene through siRNA reduces inflammatory monocytes/macrophages recruitment in ischemic areas and improves heart function in MI mice (181). Depletion of monocytes/macrophages have shown to have beneficial effects on the heart by the inhibition of deteriorating inflammatory responses. Kain et al. (135) have shown that the depletion of macrophage by clodronate liposomes significantly attenuates left ventricular hypertrophy and fibrosis and inhibits adverse cardiac remodeling in hypertensive animal models.

### 5.2 Therapeutic approaches for targeting macrophage phenotypes

Cardiac macrophages exhibit significant heterogeneity and plasticity. In general, monocyte-derived cardiac macrophage (CCR2^+^ CRM) are primarily linked to inflammatory response and pathogenic conditions, while yolk embryonic-derived CRMs (CCR2^-^ CRM) have cardioprotective effects and participate in heart reparative process (49, 182). The depletion of the entire cardiac macrophage population may result in compromised heart remodeling, due to the loss of reparative macrophages. Therefore, maintaining a delicate balance between protective and inflammatory macrophages through selective targets on macrophage phenotype is a highly attractive therapeutic strategy for treatment of heart diseases (12, 183). With the development of new biotechnologies, targeted therapies for macrophage phenotype are becoming possible.

Macrophage phenotype can be induced by cytokines, IL-1β is not only a biomarker of inflammatory macrophage but also an important cytokine to induce inflammatory macrophage differentiation (1). It has been shown that treatment with anti-IL-1β antibody or IL-1β receptor antagonist canakinumab can significantly reduce inflammation and inhibit myocardial cell apoptosis in MI animal models, while treatment with IL-4 promotes the switching of inflammatory macrophage into reparative macrophage during the inflammatory phase of AMI, thus reducing infarct size and improving pump function of the heart (184, 185).

It should be pointed out that the selective targeting or reprogramming cardiac macrophage *in vivo* for treatment of heart diseases faces many challenges, as there is a lack of unique marker to distinguish cardiac macrophage phenotypes, making it difficult to accurately target subtypes of cardiac macrophages and avoid off target effects caused by attacking other immune cells. Using *ex vivo* reprogramming autologous macrophages may be another option to overcome these shortcomings (186). For example, macrophages can be reprogrammed by incubating with M2 stimulators such as IL-10, IL-4 or TGF-β, and these reprogrammed macrophages have specific functions such as anti-inflammation, promoting angiogenesis and tissue repair, inhibiting cardiomyocyte apoptosis (187–189). Especially, autologous macrophage reprogramming *ex vivo* not only provide personalized therapy for patients, but also avoid off target macrophages. It has been demonstrated that transplantation of reprogrammed reparative macrophages induced by IL-4 or IL-10 can provide superior therapeutic benefits to the heart in mouse models of doxorubicin-induced HF and MI (190). In addition, induced pluripotent stem cell (iPSC)-derived macrophages can be generated from healthy donors, reprogramming iPSC-derived macrophages toward an anti-inflammatory phenotype are considered more effective option for treating heart diseases (191).

### 5.3 New therapeutic strategies for heart diseases based on macrophage drug delivery system

Recruitment of monocytes/macrophages and their associated inflammation are important pathogeneses of heart diseases. Since monocytes/macrophages have the ability to specifically migrate and recruit into the inflamed site, macrophages and macrophage-derived exosomes may serve as excellent drug delivery carriers (192). Nanoparticles (NPs) are formulations of diverse molecular compounds with complex architecture which are spatially organized on the scale of tens of nanometers (193). Unlike conventional pharmacotherapies with only a single therapeutic function, engineered multifunctional NPs can be designed to have ability to sense and deliver payloads, such as pharmacological activators, inhibitors, or siRNA (194). The combination of the various chemical properties of NPs and the biological targeting function of macrophages is becoming a new paradigm for the treatment of various diseases, including tumors, metabolic and cardiovascular diseases. Recently, Hu et al. (195) reported that they successfully developed a mannan-based Que@MOF/Man nanoparticles which are preferentially internalized by M2 macrophages through binding to their CD206 receptor. The administration of Que@MFO/Man exhibited favorable results in the resolution of inflammation, the prevention of adverse heart remodeling and the improvement of heart function in MI rats.

### 5.4 Clinical trials based on inhibition of macrophages or inflammation for treatment of heart disease

Current clinical trials of immunotherapy for heart diseases are mainly based on inflammatory cytokines, including IL-1β, TNFα, IL-6 and CCL2 (196). These cytokines are important biomarkers for M1 macrophages, which are also the main source of these inflammatory cytokines. Some anti-inflammatory medications or antibodies that focus on inhibiting or blocking these inflammatory cytokines have developed and clinically used for the treatment of heart diseases in clinical trials, such as MI and HF (197). A randomized and double-blind clinical trial has shown that long-term treatment with canakinumab, a therapeutic IL-1β antibody, can successfully lower inflammation and rate of recurrent cardiovascular events in the patients with post-MI (198). Colchicine is a broad-spectrum of anti-inflammatory drug that inhibits IL-1β production and TNFα-induced NF-*κ*B inflammatory pathway. Two clinical trials (COLCOT and LoDoCo2) have shown that colchicine significantly reduces ischemic cardiovascular events such as angina, MI and cardiac arrest in the patients with coronary artery diseases (199, 200). The ASSAIL-MI trial demonstrated that treatment with IL-6 antibody tocilizumab has tendency to reduce infarct size in MI patients (201). In addition, some ongoing clinical trials based on anti-inflammatory therapies have shown to reduce the incidence of major adverse cardiovascular events in the patients with coronary arterial diseases (NCT01906749 & NCT05130892) and MI (NCT02648464 & NCT01372930) (202, 203).

## 6 Conclusion and perspective

Macrophages, as a key type of immune cells residing in the heart, participate in regulating heart function in physiological condition, and induce heart remodeling in pathophysiological condition by altering their phenotypes and secreting a variety of cytokines or mediators (204). In a healthy heart, CRMs are predominated by the embryonic derived CCR2^-^ macrophages, which participate in the maintenance of heart homeostasis by clearing up apoptotic cells and cellular debris through endocytosis (77, 78). When the heart faces challenges, such as inflammation, ischemia and mechanical stress, which stimulate monocytes-derived CCR2^+^ CRMs recruitment in the heart, the cardiac macrophages undergo metabolic reprogramming to become pro-inflammatory M1 macrophage, and participate in heart ventricular remodeling under the pathological condition by releasing inflammatory cytokines and chemokines (3, 12, 111, 205). When the signal of inflammation or environment stress weakens, inflammatory M1 macrophages transform into anti-inflammatory M2 macrophages to promote heart repair.

Despite significant advancement, there are still some limitations and challenges in the field of cardiac macrophages. Firstly, there is currently no specific biomarker that can distinguish beneficial and harmful macrophage phenotypes. Current biomarkers are mainly based on cytokines and chemokines secreted by macrophages, such as CCL2, CCR2, TNFα, IL-1, IL-4 and IL-10 (119). CCR2 is the mostly used as a biomarker for distinguishing inflammatory and resident cardiac macrophages, but it is not a good molecular marker for distinguishing beneficial or detrimental macrophages (161). Some anti-inflammatory drugs or antibodies developed based on these biomarkers have entered clinical trials for immunotherapy of heart disease patients (206). However, these drugs or biological reagents cannot precisely target harmful macrophage phenotype, therefore these therapies have uncertain efficacy and side effects. Next, although the strategy targeting pathological-related cardiac macrophages or regulating function of cardiac macrophage have enormous therapeutic potential, there is currently a lack of an effective drug delivery system that specifically targets tissue or organ macrophages, especially cardiac macrophages. Finally, the current models used for *in vitro* and *in vivo* experiments of cardiac macrophages are too simple to simulate the complex pathological conditions of cardiac macrophage heterogeneity and plasticity in the patients, making it difficult to translate experimental results into clinical practice (207).

Overcoming these difficulties and challenges is our main tasks for future research on cardiac macrophages. 1) With advancement in single cell RNA-seq (scRNA-seq) and spatiotemporal transcriptomics, scRNA-seq can accurately detect and classify cell subsets, discover new cell population, and provide transcriptome profiles of cell subsets rather than just a few biomarkers (208). Spatiotemporal transcriptomics can analyze spatially separated genetic information from individual cells, and preserve their spatial location and interactions under normal heart and various pathological conditions of heart diseases (209). The application of these advanced biological technologies will enable us to precisely determine the subsets of cardiac macrophages and identify specific molecular targets on cardiac macrophages for heart diseases, particularly identifying specific molecular markers that distinguish beneficial and harmful cardiac macrophages. 2) The combination of nanoparticle drug delivery with *ex vivo* macrophage reprogramming can overcome the drawback of macrophage drug delivery systems. Nanoparticles carrying drugs, bioactive substances, or siRNA can also achieve precise targeting of cardiac macrophages, regulate macrophage function *in vivo* to achieve the goal of treating heart disease (195). Therefore, developing advanced drug delivery strategies that precisely target macrophages is expected to bring the vision of successful clinical translation to reality. 3) Long term *in vivo* studies using large animal models or humanized mice can address the translation gap between small rodents, and humans, and explore the role and phenotypic changes of macrophages over time in different cardiac pathological setting. In addition, using a culture model of precision heart slices (living myocardial slices) *ex vivo* to investigate cardiac macrophage can overcome the problem of current *in vitro* macrophage culture model being too simple (210).

It is worth noting that macrophage targeted therapies are still in the early stages of development, and these therapeutic strategies require a comprehensive evaluation of their efficacy and safety in heart disease patients. Further large-scale clinical trials may need to translate these fundamental findings into clinical practice. However, these promising therapeutic strategies are paving the way for a new era of personalized medicine based on cellular therapy, which may be applied to treat other human inflammatory diseases.
